# Mesenchymal stem cell‐inspired microgel scaffolds to control macrophage polarization

**DOI:** 10.1002/btm2.10217

**Published:** 2021-03-21

**Authors:** Alexander S. Caldwell, Varsha V. Rao, Alyxandra C. Golden, Daniel J. Bell, Joseph C. Grim, Kristi S. Anseth

**Affiliations:** ^1^ Department of Chemical and Biological Engineering University of Colorado Boulder Colorado USA; ^2^ BioFrontiers Institute, University of Colorado Boulder Colorado USA

**Keywords:** hydrogel, IL‐10, immunomodulatory, macrophage, microgel, MSC

## Abstract

There is a desire in regenerative medicine to create biofunctional materials that can control and direct cell function in a precise manner. One particular stem cell of interest, human mesenchymal stem cells (hMSCs), can function as regulators of the immunogenic response and aid in tissue regeneration and wound repair. Here, a porous hydrogel scaffold assembled from microgel subunits was used to recapitulate part of this immunomodulatory behavior. The scaffolds were used to culture a macrophage cell line, while cytokines were delivered exogenously to polarize the macrophages to either a pro‐inflammatory (M1) or alternatively activated (M2a) phenotypes. Using a cytokine array, interleukin 10 (IL‐10) was identified as one key anti‐inflammatory factor secreted by hMSCs in pro‐inflammatory conditions; it was elevated (125 ± 25 pg/ml) in pro‐inflammatory conditions compared to standard medium (6 ± 10 pg/ml). The ability of hMSC laden scaffolds to reverse the M1 phenotype was then examined, even in the presence of exogenous pro‐inflammatory cytokines. Co‐culture of M1 and M2 macrophages with hMSCs reduced the secretion of TNFα, a pro‐inflammatory cytokine even in the presence of pro‐inflammatory stimulatory factors. Next, IL‐10 was supplemented in the medium or tethered directly to the microgel subunits; both methods limited the secretion of pro‐inflammatory cytokines of encapsulated macrophages even in pro‐inflammatory conditions. Cumulatively, these results reveal the potential of biofunctional microgel‐based scaffolds as acellular therapies to present anti‐inflammatory cytokines and control the immunogenic cascade.

## 1 INTRODUCTION

Hydrogels provide a useful platform for regenerative medicine, capable of recapitulating microenvironmental cues or presenting biochemical stimuli to delivered cells.^1^, ^2^, ^3^, ^4^ In particular, the ability of scaffolds to manipulate the regenerative potential of specific stem cell types is desirable in the context of creating materials based therapies for wound healing and tissue regeneration. In this regard, human mesenchymal stem cells (hMSCs) are increasingly used in clinical trials for their reparative and regenerative potential^5^, ^6^ due to their ability to differentiate into multiple cell types.^7^, ^8^ Their role in mitigating the inflammatory response during wound healing through their secretory properties^9^, ^10^, ^11^ is particularly attractive for regenerative therapies. During periods of inflammation, endogenous hMSCs can migrate to wounds and repolarize resident inflammatory cells from inflammatory to tissue remodeling phenotypes.^12^, ^13^ Upon exposure to inflammatory cytokines (e.g., Interferon gamma (IFNγ), Interleukin 1 beta (IL‐1β), Tumor necrosis factor alpha (TNFα)), which are factors typically secreted by macrophages, hMSCs release a variety of anti‐inflammatory cytokines (e.g., TNF‐inducible gene 6 protein (TSG‐6), Transforming growth factor beta (TGFβ), Leukemia inhibitory factor (LIF), Interleukin 1 receptor antagonist (IL‐1RA))^14^, ^15^ and small molecules (e.g., Prostaglandin E2 (PGE‐2), Indoleamine 2,3‐dioxygenase (IDO), nitric oxide).^14^, ^16^ Recently, this effect has been leveraged for cell‐based therapies, where hMSCs are activated ex vivo by pro‐inflammatory conditions, a procedure termed “licensing.” Licensing is performed with the goal of manipulating hMSC secretory properties to upregulate anti‐inflammatory cytokines and ultimately, their regenerative potential.^16^, ^17^ A better understanding of the secretory profile of hMSCs and its effect on inflammatory cell function can aid in the design of acellular, functionalized materials capable of mimicking the immunomodulatory properties of hMSCs.

When designing material platforms to promote tissue regeneration, it is vital to consider the inflammatory microenvironment that exists upon injury.^18^ The inflammatory cascade is perpetuated by a variety of immune cells (e.g., neutrophils, macrophages, T cells) that arrive over different time scales.^19^ hMSCs have been demonstrated to inhibit T‐cell proliferation,^20^ modulate dendritic cell activation,^21^ limit B‐cell maturation,^22^ and direct macrophage polarization and function.^23^, ^24^ Macrophages are an innate immune cell type that help maintain tissue homeostasis with a variety of different polarization states.^25^, ^26^ However, during an acute injury response, macrophages become “activated” to an inflammatory (M1) phenotype, where they secrete a variety of pro‐inflammatory cytokines (e.g., IFNγ, TNFα, Interleukin 6 (IL‐6)) that perpetuate the immune response.^27^, ^28^ As a cell‐based therapy, hMSCs have the ability to control directly macrophage polarization, where they can reprogram M1 macrophages to a variety of different regenerative polarization states that comprise the broader M2 phenotype.^29^, ^30^ This role further aids in the resolution of chronic inflammation and improves wound healing.^31^ While the M2 phenotype encompasses several distinct phenotypes, “M2a” macrophages have both been implicated in tissue regeneration and anti‐inflammatory activity.^32^, ^33^ As macrophages infiltrate and interact with implanted biomaterials,^34^, ^35^ understanding how biomaterials can be used to modulate macrophage polarization is important for the design of effective biomaterials for in vivo applications.

While recent research has revealed several key molecules involved in the crosstalk between macrophages and hMSCs, the extent that biomaterial design can be used to alter the behavior of local and delivered cell behavior is not fully known. Clearly, biomaterial structure and composition can be modified in unique ways to influence cell behavior in the context of regenerative medicine. For example, the Geissler group demonstrated that porous materials (pore diameter ~120 μm) improve hMSC clustering and influence their regenerative effects on myoblast function.^36^ Our group expanded on this concept, by embedding hMSCs in microgel scaffolds designed to cluster hMSCs by varying the average pore size of the porous scaffold (e.g., 10 and 200 μm),^37^ where larger pore sizes upregulate the secretion of numerous trophic factors. Cell‐matrix interactions and microenvironmental structure also impact immune cell function. For example, the Bryers group demonstrated that scaffold porosity can directly impact dendritic cell function, with faster maturation occurring in scaffolds with smaller diameter (20 μm) pores compared to larger ones (90 μm).^38^ Collectively, these results point to the need to better understand the complex interplay and effects of porosity and spatial confinement on not cell function and their regenerative properties.

Beyond structural features, bioactive hydrogel scaffolds can be created through the tethering of known biochemical cues to influence cell behavior locally. For instance, Garcia et al. tethered IFNγ to hydrogel scaffolds and altered hMSC cytokine secretion, increasing the release of several immunomodulatory moieties, such as IDO and macrophage colony‐stimulating factor (M‐CSF).^39^ Conditioned medium from hMSCs cultured in IFNγ‐functionalized gels can also influence immune cell function, significantly downregulating T‐cell proliferation and dendritic cell differentiation. While stimulation (or “priming”) of MSCs is an effective way to boost their regenerative properties, the influence of this priming is often short‐lived, which can limit effectiveness. The design of acellular biomaterials to mimic this immunomodulatory potential may prove advantageous. In one early investigation, Hume et al. tethered TGF‐β1 to hydrogel scaffolds to reduce the maturation of dendritic cells in vitro.^40^ The design of similar materials that draw inspiration from the hMSC secretory profile could be highly effective in altering regeneration in vivo.

With these studies in mind, experiments herein focused on the development of porous microgel scaffolds to mimic the immunomodulatory activity of hMSCs through the direct inclusion of bioactive cues. Assembled microgel scaffolds were first designed for macrophage culture, and the macrophages were polarized to pro‐ or anti‐inflammatory phenotypes by treatment with lipopolysaccharide (LPS) and IFNγ or interleukin 4 (IL‐4) and interleukin 13 (IL‐13), respectively. hMSC immunomodulatory properties were then assessed in a context relevant to tissue regeneration in vivo, where hMSCs were co‐cultured with M1 macrophages and/or placed in pro‐inflammatory conditions (i.e., conditioned medium from M1 macrophages) to identify key immunomodulatory factors secreted by hMSCs. These factors were quantified using cytokine arrays and enzyme‐linked immunosorbent assays (ELISAs) for specific proteins. The anti‐inflammatory cytokine IL‐10 was significantly upregulated by hMSCs cultured in pro‐inflammatory conditions. hMSCs in microgel scaffolds were then co‐cultured with macrophages in separate microgel scaffolds to assess their ability to control macrophage polarization status. Based on these findings, it was investigated whether a microgel scaffold could be designed to “mimic” this MSC immunomodulatory behavior. It was hypothesized that tethering IL‐10 directly to the microgel platform would limit macrophage M1 activity, even in the presence of exogenous pro‐inflammatory cytokines. The creation of biofunctional materials that can mimic the modulatory behavior of hMSCs would be of exceptional interest to the fields of regenerative medicine.

## 2 RESULTS

### 2.1 Encapsulation and polarization of THP‐1 macrophages in clickable microgel scaffolds

**FIGURE 1:**
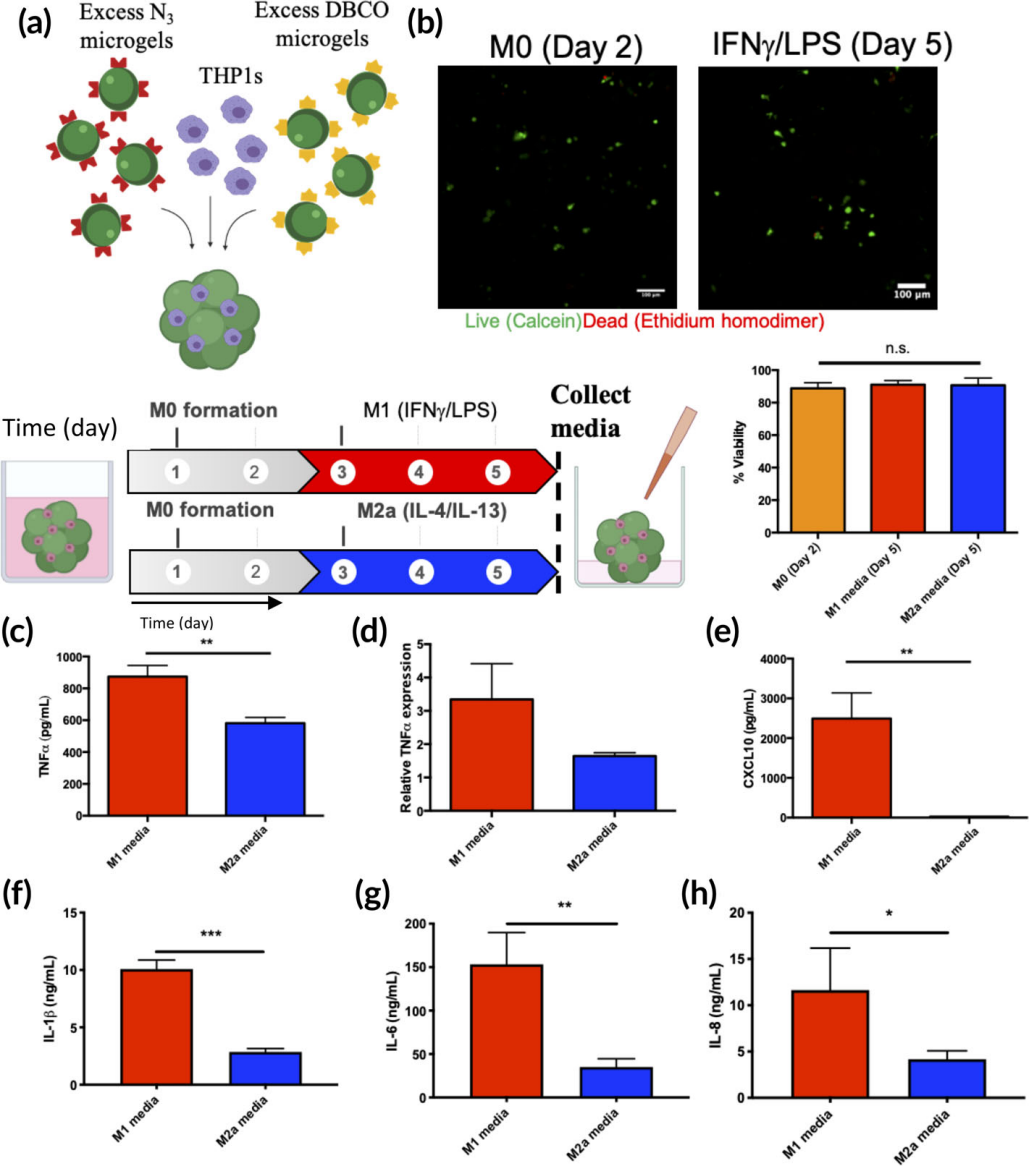
Macrophages can be encapsulated in microgel networks and polarized an inflammatory (M1) or anti‐inflammatory (M2a) phenotypes. (a) Poly(ethylene glycol) (PEG) microgel building blocks were synthesized with either excess dibenzocyclooctyne (DBCO) or N_3_ moieties and co‐assembled with THP1 cells to create porous cell‐laden scaffolds. Encapsulated THP1s were differentiated into M0 (unpolarized) macrophages over 48 h. Gels were then treated with M1 induction medium (IFNγ [10 ng/ml] + lipopolysaccharide [LPS]) or M2a induction medium (IL‐4 + IL‐13) (10 ng/ml each) for 72 h. (b) Macrophages were highly viable in microgel culture after 48 h (M0, orange bar) and after induction of the M1 (red bar) and M2a (blue bar) phenotypes. (c) Secretion and (d) mRNA expression of TNFα was significantly elevated in M1 conditions compared to M2a conditions. Secretion of the M1 markers (e) CXCL10, (f) IL‐1b, (g) IL‐6, and (h) IL‐8 was also significantly upregulated in M1 medium compared to M2a conditions.* denotes *p* < 0.05, **p < 0.01, ***p < 0.001

Clickable polyethylene glycol (PEG) microgels with excess dibenzocyclooctyne (DBCO) (8‐arm 20 kDa PEG‐DBCO) and azide (N_3_) (4‐arm 10 kDa PEG‐N_3_) surface reactive groups were fabricated and co‐assembled with THP‐1 cells to form cell‐laden porous scaffolds (average porosity ~90 μm^37^ Figure S1) (1 million cells/scaffold) (Figure 1(a)). THP‐1 s were differentiated to a macrophage phenotype (M0) over 48 h using phorbol‐12‐myristate‐13‐acetate (PMA) and subsequently polarized to pro‐inflammatory (M1) and anti‐inflammatory (M2a) phenotypes via the introduction of either IFNγ (20 ng/ml) and LPS (10 pg/ml) or IL‐4 (20 ng/ml) and IL‐13 (20 ng/ml), respectively, over 72 h. Cell viability was characterized via live/dead (calcein/ethidium homodimer) dye staining and was high after 48 h (M0s) (88 ± 4%) and 72 h later upon induction of the M1 or M2a phenotypes (M1:91 ± 3%, M2a:91 ± 5%) (Figure 1(b)). The M1 phenotype was characterized by elevated secretion of TNFα, while unpolarized and M2a polarized groups had significantly lower levels (874 ± 69 pg/ml and 581 ± 37 pg/ml, respectively) (Figure 1(c)). TNFα mRNA expression was similarly upregulated in M1 conditions compared to M2a conditions (Figure 1(d)). M1 polarized macrophages also secreted elevated levels of CXCL10 (2490 ± 650 pg/ml) compared to nearly undetectable levels in M2a conditions (30 ± 1 pg/ml) (Figure 1(e)). The pro‐inflammatory cytokines IL‐1β (10.1 ± 0.8 vs. 2.9 ± 0.3 ng/m.) (Figure 1(f)), IL‐6 (153 ± 36 vs. 35 ± 10 ng/ml) (Figure 1(g)) and IL‐8 (11.6 ± 4.5 vs. 4.2 ± 0.9 ng/ml) (Figure 1(h)) were all significantly elevated in M1 conditions compared to M2a conditions, respectively.

### 2.2 hMSCs secrete IL‐10 in response to pro‐inflammatory conditions

**FIGURE 2:**
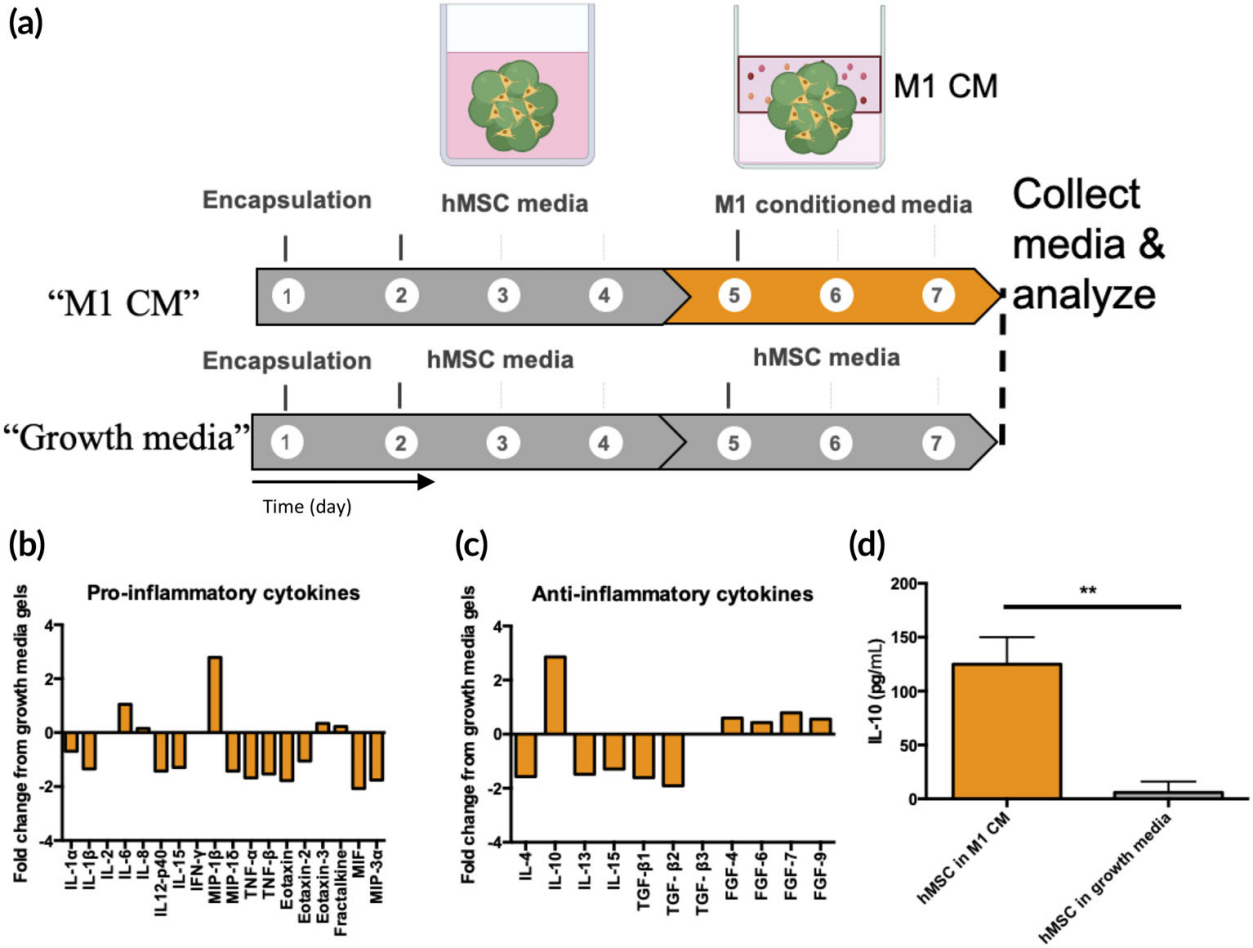
Human mesenchymal stem cells (hMSCs) secrete the anti‐inflammatory protein IL‐10 in response to inflammatory conditions. (a) hMSC laden microgel networks were cultured in a pro‐inflammatory environment (conditioned medium [CM] from M1 macrophages) to assess their secretory properties. (b) After 72 h, a cytokine array demonstrated a global decrease of many potent pro‐inflammatory factors compared to gels in standard culture conditions. (c) Analysis of several anti‐inflammatory proteins revealed that IL‐10 was highly upregulated compared to in growth medium. (d) Specific ELISA analysis demonstrated enhanced secretion of IL‐10 in response to M1 conditioned medium; **p < 0.01

After developing a microgel culture platform suitable for macrophage culture, an investigation was performed to identify relevant immunomodulatory proteins that could be incorporated into the material to control macrophage polarization. To identify factors important in modulating the macrophage phenotype, the hMSC secretory profile was assessed using a cytokine array. As previously demonstrated, larger diameter porous scaffolds promote hMSC secretory properties,^37^ so hMSCs were encapsulated in porous microgel scaffolds (particle diameter [D] = 190 ± 100 μm), and cultured in hMSC growth medium for 96 h. At that time, either fresh hMSC growth medium or a 50/50 mixture of hMSC medium and M1 conditioned medium (“inflammatory conditions”) was introduced for 72 h (Figure 2(a)). The M1 conditioned medium was generated by culturing THP1s in M1‐stimulatory medium (IFNγ/LPS) for 72 h before removing the conditioned medium and then adding it to the hMSC culture. Medium was then collected and the hMSC secretory profile was assessed via a broad screen cytokine array (RayBiotech C5 Human Cytokine Array). Compared to culture in standard growth medium, hMSCs in inflammatory conditions downregulated several potent pro‐inflammatory cytokines, including TNFα, TNFβ, and IFNγ, while there was a marked upregulation of MIP‐1β, which is chemokine that signals to a variety of immune cells (Figure 2(b)). IL‐4 and IL‐13 (M2a stimulatory factors) were downregulated by hMSCs, and several cytokines in the trophic family of fibroblast growth factors (FGF) were slightly upregulated. However, there was a significant upregulation of IL‐10 (Figure 2(c)), indicating that it may play a minor role in the reduction in the inflammatory phenotype observed previously. To further quantify this response, an ELISA was used to quantify the amount of the anti‐inflammatory cytokine with the highest fold change, IL‐10, that licensed hMSCs secreted when cultured in the pro‐inflammatory conditions (M1 conditioned medium). hMSCs secreted significantly more IL‐10 in inflammatory conditions (125 ± 25 pg/ml) compared to the growth medium condition (6 ± 10 pg/mlL) (Figure 2(d)). This is supported by previously literature identifying IL‐10 as an important immunomodulatory factor in IL‐10.^41^, ^42^

### 2.3 Control over macrophage repolarization via hMSC co‐culture

**FIGURE 3:**
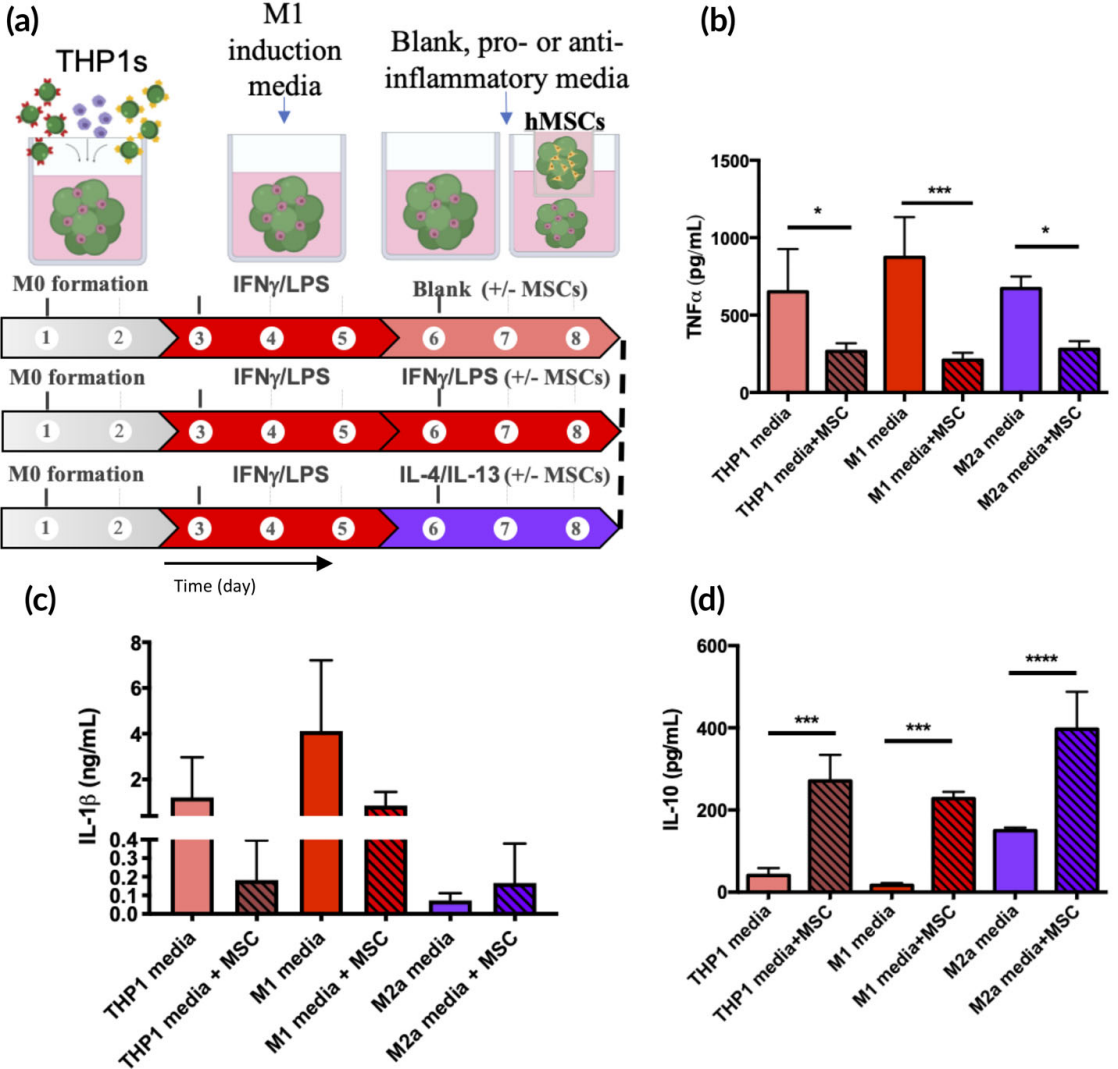
Co‐culturing human mesenchymal stem cells (hMSCs) with macrophages can limit pro‐inflammatory activity of M1 macrophages in multiple environments. (a) Macrophages were encapsulated in microgel networks and polarized to the M1 phenotype as normal over 96 h. Medium was then replaced with one of: standard THP1 medium, M1 induction medium, or M2a induction medium. Additionally, hMSCs were encapsulated in separate microgel networks and added to half of the conditions via a transwell insert. After 72 h, medium was collected and assessed for M1 and M2 specific markers. (b) TNFα secretion was significantly decreased in all M1 conditions upon co‐culture with hMSC gels, independent of medium conditions. (c) IL‐1β secretion was decreased in THP1 and M1 induction media in hMSC‐M1 co‐culture conditions compared with M1s alone and slightly upregulated in M2a medium. (d) The anti‐inflammatory cytokine IL‐10 was elevated in M2a treated conditions and was most elevated in hMSC co‐culture conditions; *p < 0.05, **p < 0.01, ***p < 0.001, n.s. = not significant (*p* > 0.05)

After examining the immunomodulatory factors secreted by hMSCs in pro‐inflammatory environments, it was next investigated whether an hMSC co‐culture could be used to reprogram the pro‐inflammatory M1 phenotype. THP‐1 macrophages were polarized to either the M1 or M2a phenotype and co‐cultured with separate hMSC‐laden microgel scaffolds (1 million cells/scaffold) (Figure 3(a)). The co‐cultures were treated with fresh THP1 growth medium or stimulatory medium for either the M1/pro‐inflammatory (IFNγ/LPS) or M2/anti‐inflammatory (IL‐4/IL‐13) condition. After 72 h, the cell medium was collected and assessed for specific M1 polarization markers (TNFα, IL‐1β). TNFα secretion remained elevated in conditions that were originally polarized to the M1 phenotype, but was significantly decreased in THP1 medium (59% decrease), pro‐inflammatory (76% decrease) and anti‐inflammatory (58% decrease) conditions when co‐cultured with hMSC‐laden gels (Figure 3(b)). IL‐1β secretion was also decreased in hMSC‐M1 co‐culture conditions compared to M1 macrophages alone in both THP1 medium (0.2 ± 0.2 vs. 1.2 ± 1.8 ng/ml) and M1 medium (0.9 ± 0.6 vs. 4.1 ± 3.08 ng/ml) but slightly increased upon exposure to M2a medium (0.2 ± 0.2 vs. 0.1 ± 0.04 ng/ml) (Figure 3(c)). Finally, the anti‐inflammatory protein IL‐10 was assessed, as it is indicative of an M2 macrophage phenotype. IL‐10 was significantly increased in hMSC‐M1 co‐culture conditions compared to M1 culture alone in standard THP1 medium (270.8 ± 63.5 vs. 40.4 ± 17.7 ng/ml), M1 medium (227.7 ± 16.7 vs. 16.8 ± 5.6 ng/m.) and M2a medium (396.8 ± 90.8 vs. 149.8 ± 7.7 ng/m.) (Figure 3(d)). Overall, hMSC co‐culture reduced THP1 M1 macrophage polarization, evidenced by a reduction in the secretion of M1 characteristic cytokines, even in the presence of pro‐inflammatory stimulatory proteins.

### 2.4 Effects of IL‐10 on macrophage polarization

**FIGURE 4:**
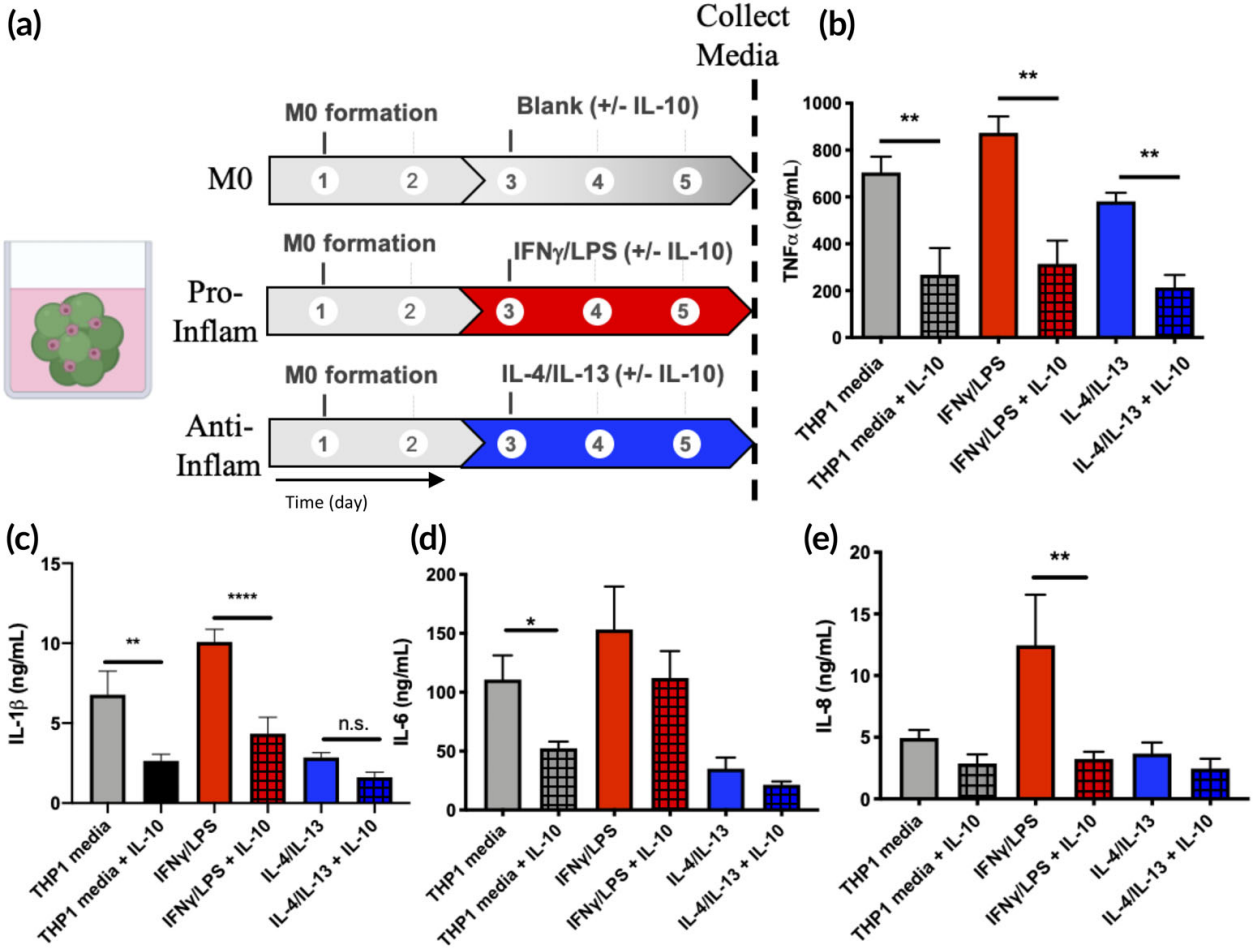
IL‐10 reduces the secretion of pro‐inflammatory cytokines by THP1 macrophages in both pro‐ and anti‐inflammatory environments. (a) M0 THP1s encapsulated in microgel scaffolds were treated with or without exogenous IL‐10 in THP1 medium, pro‐(IFNγ/LPS) or anti‐inflammatory (IL‐4/IL‐13) conditions. After 72 h of exposure to IL‐10, medium from each condition was saved and analyzed for the presence of pro‐inflammatory cytokines. (b) TNFα secretion was significantly decreased in all IL‐10 treated conditions compared to their corresponding untreated condition. (c) IL‐1β was significantly reduced in IL‐10 treated networks in THP1 medium and pro‐inflammatory conditions, and nonsignificantly reduced in IL‐10 treated gels in anti‐inflammatory conditions compared to untreated networks. (d) IL‐6 was significantly reduced in IL‐10 treated networks in THP1 medium conditions, and nonsignificantly reduced in IL‐10 treated networks in pro‐ and anti‐inflammatory conditions compared to untreated networks. (e) IL‐8 was significantly reduced in IL‐10 treated networks in pro‐inflammatory conditions, and nonsignificantly reduced in IL‐10 treated gels in THP1 medium or anti‐inflammatory conditions compared to untreated networks; *p < 0.05, **p < 0.01, ***p < 0.001, n.s. = not significant (*p* > 0.05) for one‐way ANOVA with multiple comparisons between groups

Given IL‐10 was upregulated by hMSCs cultured in inflammatory conditions and that hMSC co‐cultures limited M1 macrophage activity, the effect of local presentation of IL‐10 on macrophages was subsequently investigated. First, a dose screen was performed using THP1 macrophages cultured on tissue culture polystyrene (TCPS), and 10 ng/ml of IL‐10 significantly reduced TNFα secretion (Figure S2). Next, macrophages were encapsulated and left unpolarized (M0 condition) or placed in pro‐(IFNγ/LPS) or anti‐inflammatory (IL‐4/IL‐13) conditions in the presence or absence of 10 ng/ml of IL‐10 (Figure 4(a)). After 72 h of culture with or without exogenous IL‐10, medium from each gel was collected and the amount of pro‐inflammatory cytokines (TNFα, IL‐1β, IL‐6, IL‐8) measured. IL‐10 treatment significantly decreased TNFα secretion by encapsulated macrophages in the THP1 medium, pro‐ and anti‐inflammatory conditions compared to untreated controls (Figure 4(b)). TNFα secretion decreased from 705 ± 67 to 268 ± 114 pg/ml in THP1 medium, from 874 ± 70 to 314 ± 99 pg/ml in pro‐inflammatory conditions, and from 581 ± 37 to 214 ± 53 pg/ml in anti‐inflammatory medium upon the addition of IL‐10. Secretion of IL‐1β was also significantly affected by IL‐10 treatment, with significant decreases in both THP1 medium (from 6.79 ± 1.47 to 2.64 ± 0.42 ng/ml) and pro‐inflammatory conditions (from 10.08 ± 0.78 to 4.35 ± 1.02 ng/ml), and a nonsignificant decrease in anti‐inflammatory conditions (from 2.86 ± 0.30 to 1.62 ± 0.31 ng/ml) (Figure 4(c)). IL‐6 levels were also decreased in all conditions upon treatment with IL‐10 (from 111 ± 21 to 52 ± 6 pg/ml in THP1 medium, from 153 ± 36 to 112 ± 23 pg/ml in pro‐inflammatory conditions and from 35 ± 10 to 21 ± 3 pg/ml in anti‐inflammatory conditions), though only significantly in the THP1 medium condition (Figure 4(d)). Finally, the secretion of IL‐8 was assessed, with levels decreasing from 4.95 ± 0.65 to 2.87 ± 0.74 ng/ml in THP1 medium, from 12.47 ± 4.09 to 3.25 ± 0.58 ng/ml in pro‐inflammatory conditions and from 3.67 ± 0.90 to 2.45 ± 0.81 ng/ml in anti‐inflammatory conditions with treatment of IL‐10 (Figure 4(e)).

**FIGURE 5:**
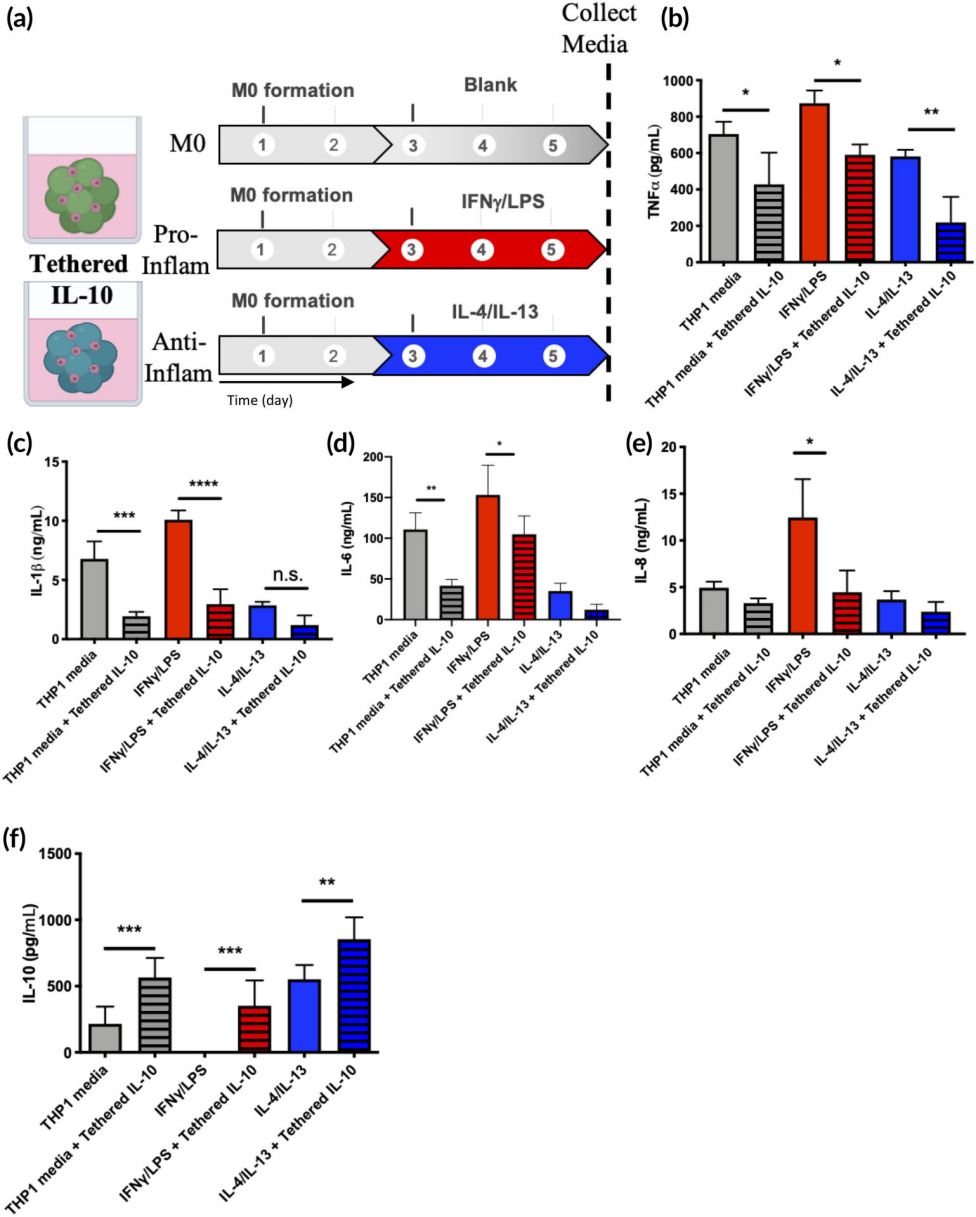
Tethered IL‐10 microgel scaffolds reduces the secretion of pro‐inflammatory activity by THP1a. (a) THP1s were then encapsulated within unmodified or IL‐10 tethered microgel scaffolds and polarized to pro‐ or anti‐inflammatory conditions as normal. (b) TNFα secretion was significantly decreased in all IL‐10 tethered network conditions compared to the corresponding unmodified networks. (c) IL‐10 networks significantly reduced IL‐1β in THP1 medium and pro‐inflammatory conditions, and nonsignificantly reduced in tethered IL‐10 networks in anti‐inflammatory conditions. (d) IL‐6 was also significantly reduced in THP1 medium and proinflammatory conditions, and nonsignificantly reduced in tethered IL‐10 networks in anti‐inflammatory conditions. (e) IL‐8 was significantly reduced in pro‐inflammatory conditions and nonsignificantly reduced in tethered networks in both THP1 medium and anti‐inflammatory conditions. (f) Finally, IL‐10 secretion by macrophages was significantly elevated in IL‐10 gels compared to their unmodified counterparts. *p < 0.05, **p < 0.01, ***p < 0.001, ****p < 0.0001, n.s. = not significant (*p* > 0.05) for one‐way ANOVA with multiple comparisons between groups

Based on these data, an azide‐modified IL‐10 was synthesized via an NHS coupling reaction. The modified IL‐10 was tethered directly to the microgel subunits during fabrication at 10 ng/ml and confirmed by immunostaining (Figure S3). THP1 cells were then encapsulated within the IL‐10 modified microgel scaffolds (Figure 5(a)). Compared to unmodified scaffolds, TNFα secretion from macrophages was significantly decreased in the IL‐10‐functionalized scaffolds for all conditions (THP1 medium: from 705 ± 67 to 428 ± 174 pg/ml, pro‐inflammatory: from 874 ± 70 to 590 ± 57 pg/ml, and anti‐inflammatory: 581 ± 37 to 219 ± 141 pg/ml) (Figure 5(b)). Similarly, IL‐1β levels were decreased in IL‐10 tethered scaffolds (THP1 medium: from 6.79 ± 1.47 to 1.93 ± 0.38 ng/ml, pro‐inflammatory: from 10.08 ± 0.78 to 2.95 ± 1.27 ng/ml and anti‐inflammatory: 2.86 ± 0.30 to 1.19 ± 0.81 ng/ml [nonsignificant]) compared to control scaffolds (Figure 5(c)).. IL‐6 levels were significantly decreased in tethered IL‐10 gels compared to unmodified gels in both THP1 medium (from 111 ± 21 to 42 ± 8 pg/ml) and pro‐inflammatory conditions (from 153 ± 37 to 105 ± 22 pg/ml), and nonsignificantly decreased in anti‐inflammatory conditions (from 35 ± 10 to 12 ± 7) (Figure 5(d)). The secretion of IL‐8 was significantly reduced in IL‐10 tethered gels in pro‐inflammatory environments (from 12.47 ± 4.1 to 4.45 ± 2.34 ng/ml), and nonsignificantly in both THP1 medium (from 4.94 ± 0.65 to 3.29 ± 0.52 ng/ml) and anti‐inflammatory conditions (3.67 ± 0.90 to 2.37 ± 1.05 ng/ml) (Figure 5(e)). Finally, elevated levels of IL‐10 (indicative of a more M2‐phenotype) were detected in culture medium, despite negligible release from acellular IL‐10 modified scaffolds (Figure S4). Macrophages encapsulated in IL‐10 tethered gels secreted significantly higher levels of IL‐10 compared to those in unmodified networks in THP1 medium (from 216 ± 129 to 565 ± 147 pg/ml), pro‐ (from undetectable levels to 352 ± 191 pg/ml), and anti‐inflammatory (552 ± 107 to 854 ± 165 pg/ml), environments (Figure 5(f)). Overall, IL‐10 modified microgel scaffolds were able to significantly reduce THP1 inflammatory activity, even in the presence of exogenous pro‐inflammatory factors.

**FIGURE 6:**
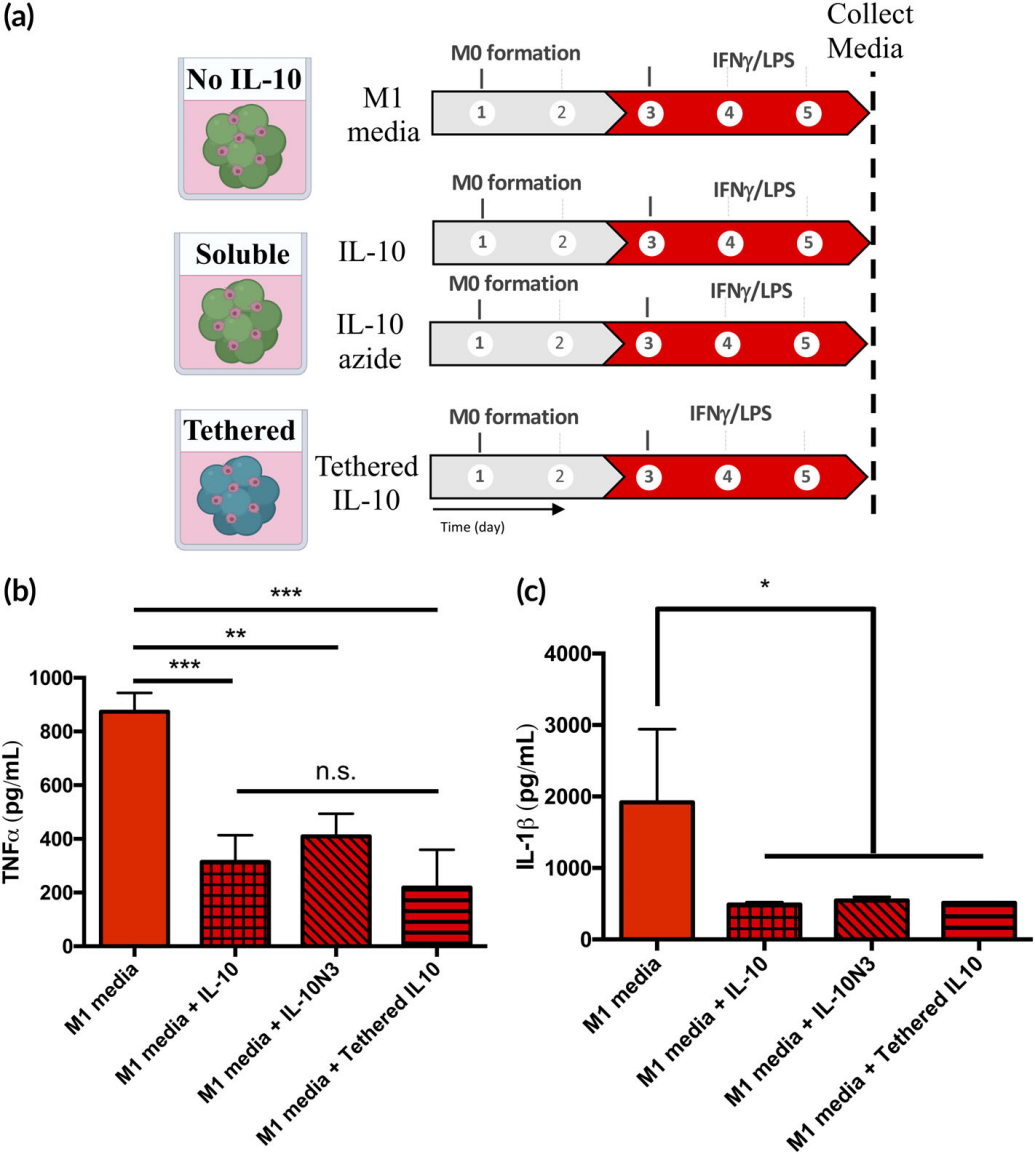
Azide‐modified and tethered IL‐10 result in similar changes in macrophage phenotype as unmodified IL‐10. (a) Microgel scaffolds were treated with M1 induction media along with 10 ng/ml IL‐10 or IL‐10‐N_3_ and compared to IL‐10 tethered microgel scaffolds. (b) TNFα secretion was reduced in all IL‐10 conditions with no significant difference between the soluble, azide modified, and tethered groups. (c) The secretion of the pro‐inflammatory cytokine IL‐1β was significantly decreased upon treatment with 10 ng/ml IL‐10, IL‐10‐N_3_ and in IL‐10 tethered microgel scaffolds in M1 induction conditions. **p < 0.01, ***p < 0.001, n.s. = not significant (*p* > 0.05)

Finally, it was assessed whether the effects of tethered IL‐10 on macrophage pro‐inflammatory activity were comparable with those of soluble IL‐10 (Figure 6(a)). Specifically, levels of macrophage secreted TNFα and IL‐1β were assessed by macrophages in either scaffold in the presence of M1 stimulatory cytokines. TNFα secretion was significantly downregulated for macrophages in M1 media when exposed to IL‐10, but there was no significant difference between those treated solubly with IL‐10, azide‐modified IL‐10, or those in IL‐10 tethered microgel scaffolds (Figure 6(b)). Similar results were observed for IL‐1β, with no significant change in macrophage secretion when exposed to IL‐10 in each of the three forms in M1 media (Figure 6(c)). This indicates that neither the modification of IL‐10 with the azide handle nor the tethering to the microgel scaffold severely impacted its bioactivity.

## 3 DISCUSSION

As the field of regenerative medicine progresses, there is an increasing need for material platforms that can modulate and control the immune response. Chronic inflammation, promoted through M1 macrophages, can impede proper wound healing,^43^, ^44^ so the resolution of the inflammatory cascade (e.g., M1 to M2 macrophage polarization) is the focus of many biomaterial and cell based therapies aimed at promoting tissue regeneration. Indeed, hMSCs have been used in numerous pre‐clinical and clinical trials,^45^ with many of the therapeutic effects attributed to their ability to signal to endogenous cells to stimulate tissue regeneration.^46^, ^47^ Specifically, hMSCs are known to modulate immune cells, both in vivo and in vitro,^48^, ^49^, ^50^ and as we demonstrate, they have the unique ability to shift macrophage polarization from an activated M1 phenotype to a deactivated “M2” phenotype.^30^, ^51^ While in reality the M2 phenotype represents a broad range of macrophages with distinct functions,^25^ the M1‐M2 paradigm remains an effective, albeit simplistic, manner to view their respective roles in inflammation. As hMSCs show the potential for transplantation into inflammatory environments to modulate the local immune response, examining their immunomodulatory potential can help to inform biomaterial design that can mimic this behavior.^52^, ^53^ In this study, hMSC laden microgels were cultured in the presence of prepolarized M1 macrophages and found to limit activation to the M1 phenotype, even in the presence of pro‐inflammatory stimuli. Specifically, hMSCs downregulated the macrophage's production of the pro‐inflammatory cytokines TNFα and IL‐1β. Our modular cell culture platform allows us to investigate the immunomodulatory potential of hMSCs in response to pro‐inflammatory cell activity in a 3D culture environment. Deeper investigation of the immune‐regulatory function of hMSCs in response to high levels of pro‐inflammatory factors^14^ can be used to design and engineer acellular biomimetic scaffolds for regenerative therapies.

As the immunomodulatory potential of hMSCs is modulated by environmental stimuli, we set out to understand the effects of pro‐inflammatory macrophages on the hMSC secretome. Co‐culture models can complicate determination of the source of cytokine production from the two cell types, as hMSCs can also secrete a wide variety of cytokines.^48^ To circumvent this, hMSCs were first cultured in M1 conditioned medium (pro‐inflammatory conditions) to isolate their secretory properties from that of the macrophages. The hMSC secretory properties changed dramatically in pro‐inflammatory conditions, downregulating a variety of pro‐inflammatory cytokines and upregulating IL‐10, as measured by a broad screen cytokine array. As we observed a decrease in pro‐inflammatory activity of macrophages co‐cultured with hMSCs, we focused on the specific role of the main upregulated anti‐inflammatory cytokine, IL‐10. This observation was supported by a body of literature that has implicated IL‐10 in immunomodulatory properties of hMSCs as well as the M2 phenotype.^10^, ^11^, ^54^, ^55^, ^56^ IL‐10 was incorporated into macrophage cultures at a concentration of 10 ng/ml, which is equivalent to the concentration of the other M1/M2a stimulatory factors (e.g., IFNγ, IL‐4, IL‐13). Interestingly, similar reductions in TNFα secretion were observed by macrophages in IL‐10 treated conditions as in hMSC co‐culture conditions, despite the fact that concentrations of hMSC‐secreted IL‐10 were two orders of magnitude lower. This supports the notion that there are numerous cytokines and small molecules involved in hMSC immunomodulatory activity.^10^, ^14^, ^48^ Continuing efforts are focused on understanding controlling hMSC regulatory potential in precise manners, specifically by using material‐design strategies to alter these secretory properties and to develop acellular materials that can capture key aspects of their regenerative properties.^2^, ^39^, ^57^, ^58^ Focus on measuring other hMSC‐secreted proteins, small molecules, and exosomes, as well as how their specific levels change in response to specific culture conditions, will likely help better define the collective and/or synergistic potential of the hMSC immunomodulatory activity.

While biochemical stimuli,^59^ porosity,^34^, ^60^, ^61^ and polymer chemistry^62^, ^63^ have all been tailored to modulate immune cell‐biomaterials interactions, we specifically explored the ability of IL‐10‐functionalized scaffolds to modulate macrophage polarization. While cell‐based therapies provide promise for regulating regeneration, there are also significant drawbacks (e.g., variability in therapeutic efficacy, regulatory issues, low cell survivability).^64^, ^65^, ^66^ Acellular biomaterials platforms that could replicate aspects of the immunomodulatory behavior of hMSCs may prove advantageous for the future of regenerative medicine.^67^, ^68^ We demonstrate that IL‐10 is able to reduce macrophage pro‐inflammatory activity, both when introduced solubly in the medium and when tethered directly to microgel scaffolds using a SPAAC reaction. Macrophages encapsulated in IL‐10 functionalized scaffolds had reduced pro‐inflammatory activity, similar to macrophages exposed to soluble IL‐10, supporting the notion that the protein is stable after conjugations. Future studies should further investigate this concept, specifically considering changes in the duration of IL‐10 signaling to cells. It has been documented that the context of a biomolecules presentation (i.e., soluble or tethered) can greatly impact its stability or cellular signaling.^39^, ^69^, ^70^ Any extension in the lifetime of cue signaling would highlight the potential regenerative impact of these bioactive acellular materials. To fully explore the potential of these scaffolds, translation to an in vivo setting is needed. The in vitro studies presented here provide a highly specified environment for understanding cell‐material interactions in a precise manner. The demonstration that an acellular platform can provide similar anti‐inflammatory stimuli to macrophages as hMSCs is a significant demonstration and holds promise for future translational applications. In total, we anticipate that the development of our in vitro platform that allows for probing of immune cell polarization and reprogramming in response to matrix interactions in precise manners should aid in the design of scaffolds for regenerative therapies.

The field of regenerative medicine has repeatedly emphasized how innovative material design strategies are needed to control cell function in precise manners.^3^, ^71^, ^72^ Both biochemical (e.g., anti‐inflammatory cytokines or small molecules)^1^, ^40^ and structural (e.g., network porosity)^38^ cues can be incorporated to modulate immune cell function in multiple aspects simultaneously. Based on these results, a systematic and high‐throughput screening of the effects of scaffold porosity and network structure on macrophage polarization could be an interesting line of query. The microgel‐based scaffold employed here design would allow for independent tailoring of these variables, as well as a facile route to the incorporation of multiple cues simultaneously. The development of materials that are capable of delivering of multiple cytokines simultaneously to control macrophage polarization may improve both cellular and acellular regenerative therapies and wound healing. Deeper analysis of hMSC immunomodulatory properties can help inform this work by incorporating other cytokines or small molecules implicated in this response (e.g., TGFβ, HGF, PGE_2_) into our biomaterial design.^14^ A combinatorial approach using multiple factors can improve the effectiveness of these instructive microgel scaffolds in reprogramming macrophage polarization. Similarly, more extensive analysis of the timescales of inflammation in the wound healing process could inform material design, where anti‐inflammatory presentation can be designed to match these timelines. Engineering biomaterials that can control the immune response directly can improve translational medicine and pave the way for acellular regenerative therapies.

## 4 MATERIALS AND METHODS

### 4.1 Macromer synthesis and microgel fabrication

Briefly, eight‐arm poly(ethylene glycol) (PEG) amine hydrochloride (JenKem, 20,000 g/mol) was functionalized with dibenzocyclooctyne (DBCO) as previously reported.^73^ PEG‐azide (PEG‐N_3_) (four‐arm, 10,000 g/mol) was also synthesized as previously described.^74^ End‐group functionalization was verified via ^1^H NMR. An azide functionalized cellularly adhesive peptide (K(N_3_)GRGDS) (arginylglycylaspartic acid or “RGD”) was synthesized using standard Fmoc chemistry using a Protein Technologies Tribute Peptide Synthesizer on a Rink Amide MBHA resin. K(N_3_) refers to the azide‐l‐lysine (ChemImpex). RGD was purified using reverse phase high pressure liquid chromatography (HPLC) and analyzed using electrospray ionization (ESI) mass spectroscopy.

Microgels were fabricated using an inverse suspension polymerization as previously described.^73^ Briefly, PEG‐DBCO, PEG‐N_3_, and RGD‐N_3_ (azide labled RGD) were combined and rapidly transferred to a hexane solution with Span‐80 (2.25%/volume) and Tween‐20 (0.75%/volume). Solutions were exposed to high (vortexing) or low (magnetic stirring) shear for 5 min to allow for complete polymerization. Full characterization of these particle sizes and mechanics has been previously reported.^37^ High shear resulting in microgels with an average diameter of 110 ± 60 μm (used for THP1 encapsulation) and low shear resulting in microgels with an average diameter of 190 ± 100 μm (used for hMSC encapsulation). Two distinct microgel populations were formed with 11 mM excess of either functional group (DBCO or N_3_) to allow for subsequent cross‐linking upon assembly into a larger scaffold. After formation, microgels were washed with isopropanol (4x) and sterile phosphate buffered saline (PBS) before resuspension in PBS.

### 4.2 Microgel scaffold formation and cell encapsulation

Cell‐laden microgel scaffolds were formed as previously described.^37^, ^73^ About 50 μl (preswollen volume) of each microgel population (DBCO or N_3_ excess) were combined into 2 ml of PBS in sterile 15 ml conical tubes. The 1 million cells (either THP‐1 or hMSCs) were added to this suspension to create cell laden scaffolds. THP‐1s were encapsulated in 110 ± 60 μm diameter microgel scaffolds, while hMSCs were encapsulated in 190 ± 100 μm diameter microgel scaffolds. Microgel suspensions were centrifuged for 10 min at 1000*g* to allow for complete scaffold assembly. Microgel scaffolds were removed and immediately placed in 1 ml of corresponding cell medium. For all co‐culture experiments, transwell inserts (Milipore Sigma) were used to maintain the same medium for both gels. Particle size, network porosity and pore dimensions were assessed using a previously reported Matlab script.^73^ Briefly, particles were labeled with azide‐ AlexaFluor 647 (final concentration = 40 μm, Life Technologies) and imaged using a laser scanning confocal (Zeiss LSM710). Representative images of particle sizes and scaffold porosity are shown in Figure S1.

### 4.3 Cell culture

Human mesenchymal stem cells (hMSCs) were isolated from fresh human bone marrow (Lonza, donor 18‐year‐old black female) as previously published.^75^ Upon isolation, cells were frozen in cell‐freezing medium (ThermoFisher) and stored in liquid nitrogen until use. All experiments used hMSCs at passage 3. hMSC medium consisted of low glucose (1 g/L) Dulbecco's Modified Eagle Medium (ThermoFisher) with 10% FBS (ThermoFisher), 50 U/ml penicillin (ThermoFisher), 50 μg/ml streptomycin (ThermoFisher), and 0.5 μg/ml of amphotericin B (ThermoFisher).

The human monocytic leukemia cell line THP‐1 (ATCC) was cultured according to ATCC protocol. THP‐1 medium consisted of RMPI (ThermoFisher) medium supplemented with 10% FBS (ThermoFisher), 50 U/ml penicillin (ThermoFisher), 50 μg/ml streptomycin (ThermoFisher), 0.5 μg/ml of amphotericin B (ThermoFisher), and 2‐mercaptoethanol (50 μM). Cells were used between passage 5 and 15 for all experiments.

THP‐1 polarization was based on previously published protocols.^76^ After encapsulation, cells were treated with THP‐1 medium supplemented with phorbol 12‐myristate 13‐acetate (PMA) (0.15 μM) to differentiate the THP1s into M0 macrophages. After 24 h, M0 medium was replaced with fresh THP‐1 medium. After 24 h, cells were polarized into M1 or M2a states over 72 h with specific induction cues. Cells were given fresh THP‐1 medium (nonpolarized), IFNγ (20 ng/ml), and lipopolysaccharide (LPS) (0.01 ng/ml) (M1 polarization), or IL‐4 (20 ng/ml) and IL‐13 (20 ng/ml) (M2a polarization). For all repolarization experiments, gels were washed twice with fresh THP‐1 medium at the end of the polarization period and placed in new polarization medium for 72 h.

IL‐10 dosing was determined via a concentration screen using THP1s cultured on tissue culture polystyrene (TCPS). THP1s were cultured at 20,000 cells/cm^2^ and polarized to the M1 phenotype in the presence of 0.1, 1 or 10 ng/ml human recombinant IL‐10 (R&D systems) (Figure S2). Medium was assessed for TNFα secretion as described below.

### 4.4 Macrophage polarization analysis

Macrophage medium was collected at the end of polarization or repolarization and saved for specific protein analysis. ELISAs (R&D systems “Duo” kits for TNFα, IL‐1β, IL‐6, IL‐8, IL‐10) were performed according to the manufacturer's protocols.

RNA isolation was performed using a RNeasy mini kit (Qiagen) according to the manufacturer's protocol. RNA concentration was determined using an ND‐1000 Nanodrop Spectrophotometer. For RT‐qPCR, cDNA was synthesized via the iScript Reverse Transcription Supermix kit (Bio‐Rad) using an Eppendorf Mastercycler. Custom primers were designed and presented in Table S5. Relative mRNA expression levels were quantified using SYBR Green reagent (Bio‐Rad) on an iCycler machine (Bio‐Rad). Normalization was performed relative to the housekeeping gene GAPDH.

### 4.5 Preparation of macrophage conditioned medium and culture of hMSCs in pro‐inflammatory environments

Macrophage conditioned medium was prepared from M1 macrophages plated on 2D tissue culture polystyrene. THP‐1 cells were plated at a cell density of 60,000 cells/cm^2^ and cultured with 1 ml of medium per 30,000 cells. Macrophages were polarized to an M1 phenotype using the previously mentioned protocol.

hMSCs were encapsulated in microgel scaffolds and cultured for 24 h in standard hMSC medium. Subsequently, gels were cultured in a mixed medium containing 0.5 ml of hMSC medium and 0.5 ml of macrophage conditioned medium. After 3 days, medium was removed and saved for analysis via ELISA, while gels were homogenized (TissueLyser II, Qiagen, 30 Hz for 1 min) and digested in papain solution (1 mg/ml, Sigma) in PBE buffer with 1.77 mg/ml l‐cystein overnight at 65°C. DNA amount per gel was determined using a Quant‐It PicoGreen assay (ThermoFisher). The global secretory profile of the hMSCs was assessed using a Human Cytokine Array (C5, RayBiotech) according to the standard protocol. Briefly, arrays were blocked and incubated with hMSC gel medium overnight at 4°C. Arrays were washed, incubated with a biotinylated antibody cocktail for 2 h (room temperature), washed, and labeled with an HRP‐strepdavidin solution for 2 h (room temperature). Gels were then washed and incubated with the provided detection buffer and imaged on an ImageQuant LAS 4000 (GE Healthcare) to quantify chemiluminescence. Raw data were analyzed (ImageQuant) and values were normalized per the manufacturer's protocol. Final values were normalized to readings from an array with unmodified hMSC medium (to account for any cytokines present in the added 10% FBS) and to μg DNA as determined by PicoGreen assay.

### 4.6 Tethering of IL‐10 to microgel networks

Human IL‐10 (R&D systems) was reconstituted at 50 μg/ml according to the manufacturer's specifications. 1.8 μl of NHS‐PEG_4_‐Azide (4 mM in DMSO, ThermoFisher Scientific) was added to 90 μl of IL‐10 solution and coupling was performed for 2 h at 4°C. Protein was diluted to 200 μl and dialyzed for 2 h at 4°C in 0.1% BSA in PBS. Protein incorporation was achieved at 10 ng/ml by including azide‐IL‐10 in the microgel formulation. This corresponds to an IL‐10 concentration of approximately 18.2 ng/mm^2^ considering particle size. To confirm protein modification, bulk gels with excess DBCO moieties were fabricated with AlexaFluor 647 (40 μm) and either unmodified IL‐10 or IL‐10‐N_3_. After fabrication, gels were swollen in PBS for 72 h at room temperature. Gels were then washed with PBS containing 0.05% Tween‐20 (3x, 1 h each), blocked with 5% bovine serum albumin (BSA) for 1 h and immunostained using a monoclonal antibody for IL‐10 (Abcam) (1:200 in 5% BSA) overnight at 4°C. Gels were washed with PBS (0.05% Tween‐20) three times, and incubated with goat anti‐rabbit AlexaFluor 488 (1:300 dilution) in 5% BSA (Figure S3). To evaluate release of any untethered IL‐10, acellular microgel scaffolds were fabricated with 10 ng/ml of either IL‐10 N_3_ or unmodified IL‐10 and placed in THP‐1 medium. All media was collected and replaced 24, 48, and 120 h after encapsulation an assessed using an IL‐10 ELISA according to manufacturer's protocol (R&D systems), with no observable difference in assay reactivity to identical concentrations stock solutions IL‐10 N_3_ or unmodified IL‐10 (Figure S4).

### 4.7 Statistical analysis

Statistical analysis and interpolation for specific ELISAs was conducted using GraphPad Prism software. Statistical significance was determined using one‐way ANOVAs with multiple post hoc comparisons (Tukey correction). All data represent three independent biological replicates unless noted otherwise. All data are presented as the mean value plus/minus SD unless otherwise stated.

## 5 CONCLUSION

In the presented work, microgel assembled scaffolds were designed to modulate macrophage polarization directly. THP‐1 cells were encapsulated with microgel networks and polarized to M1 and M2a phenotypes. Macrophage phenotype was reprogrammed through the introduction of exogenous cytokines, and this repolarization was further improved through hMSC co‐culture conditions. Further investigation of the immunomodulatory potential of hMSCs revealed a distinct secretory phenotype in inflammatory conditions and revealed IL‐10 as a key mediator of the reprogramming of macrophages. Finally, IL‐10 was introduced into macrophage culture, both solubly and tethered to microgel subunits, to direct macrophage polarization. These results can be used to further inform biomaterial design in regenerative medicine, both for cell transplantation efforts and acellular biomaterial implantation.

## CONFLICT OF INTERESTS

The authors declare no conflicts of interest.

## AUTHOR CONTRIBUTIONS

**Alexander Caldwell:** Conceptualization; data curation; formal analysis; funding acquisition; investigation; methodology; project administration; validation; writing‐original draft; writing‐review & editing. **Varsha Rao:** Conceptualization; data curation; formal analysis; funding acquisition; investigation; project administration; writing‐original draft; writing‐review & editing. **Alyxandra Golden:** Data curation; formal analysis; writing‐review & editing. **Daniel Bell:** Data curation; formal analysis; methodology; writing‐review & editing. **Joseph Grim:** Conceptualization; writing‐review & editing. **Kristi Anseth:** Conceptualization; funding acquisition; project administration; resources; supervision; writing‐review & editing.

### PEER REVIEW

The peer review history for this article is available at https://publons.com/publon/10.1002/btm2.10217.

## Supporting information


**Appendix S1**: Supporting Information

## Data Availability

Data Availability: The raw data used in this study are available from the corresponding author upon reasonable request.
