# Supplementary material for: Mesenchymal stem cell‐inspired microgel scaffolds to control macrophage polarization
Source: Bioeng Transl Med. 2021 Mar 21;6(2):e10217. doi: 10.1002/btm2.10217 (PMC8126823; doi:10.1002/btm2.10217)

**Mesenchymal Stem Cell-Inspired Assembled Hydrogel Scaffolds to Control Macrophage Polarization**

Alexander S Caldwell^1,2^, Varsha V Rao^1,2^, Alyxandra C Golden^1^, Daniel J Bell^1,2^,

Joseph C Grim^1,2^, Kristi S Anseth^1,2^

Affiliations: 1: Department of Chemical and Biological Engineering, University of Colorado, Boulder

2: BioFrontiers Institute, University of Colorado, Boulder

All correspondence can be directed to K.S. Anseth at kristi.anseth@colorado.edu

Keywords: Microgel, Hydrogel, MSC, Macrophage, Immunomodulatory, IL-10

**Supplementary information**

**
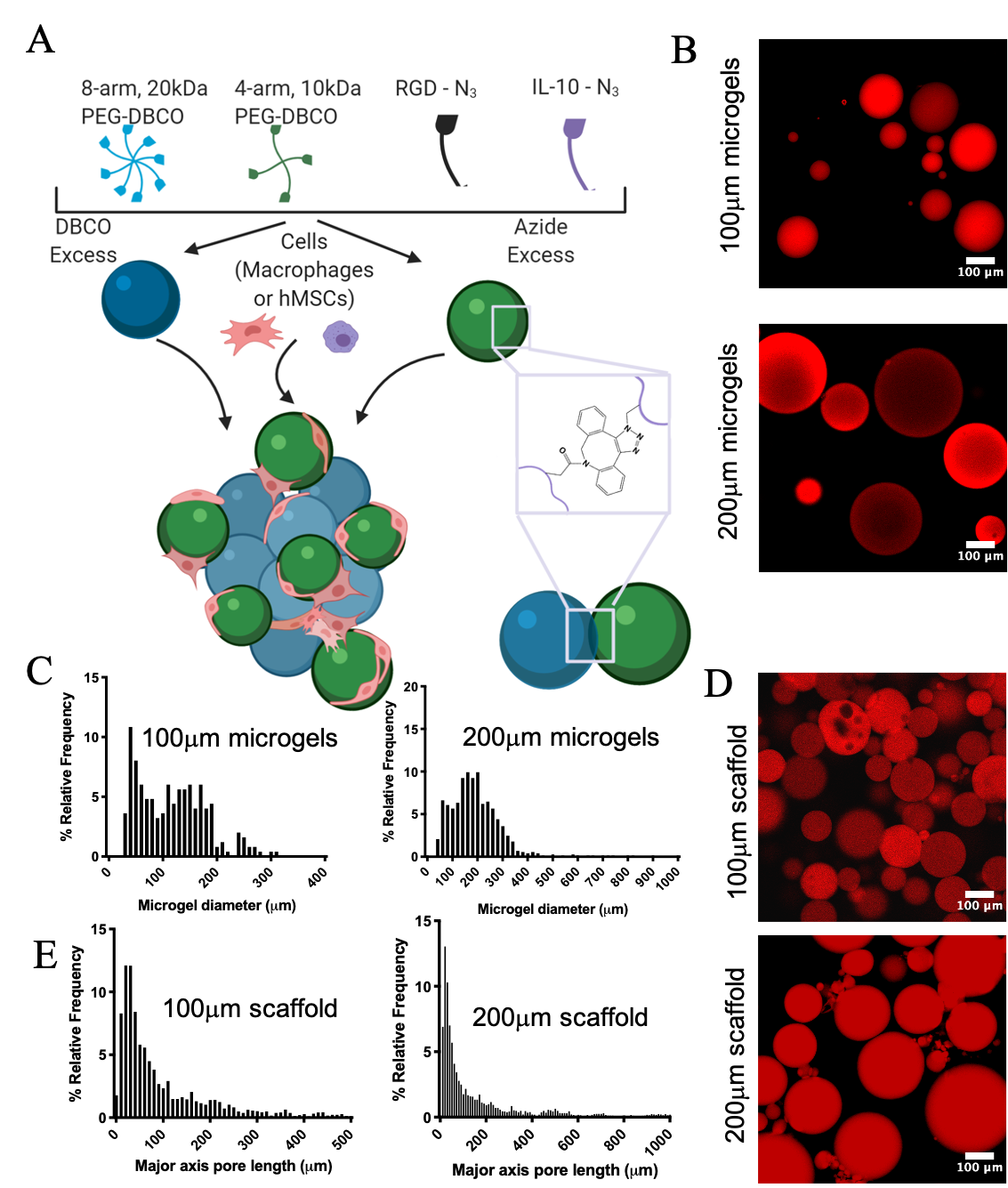
**

**Figure S1: Formulation and characterization of microgel assembled scaffolds**. A) Microgels were formulated with excess DBCO or N_3_ groups and assembled into porous cell culture scaffolds. Microgels were fabricated with the adhesive peptide RGD, and IL-10 functionalized scaffolds were created by incorporating IL-10 into the microgel formulations. B) Particles with average diameters of ~100μm and ~200μm were fabricated and visualized with AlexaFluor 647 azide. C) Matlab image analysis was used to quantify particle diameters. D) The two particle populations were assembled into porous scaffolds, visualized and E) their pore dimensions quantified via image analysis.

**Figure S2: TNFα secretion of M1 polarized THP1s in the presence of IL-10.** THP1s were cultured on TCPS at 20,000 cells/cm^2^ and polarized to the M1 phenotype with standard M1 media, or M1 media supplemented with 0.1, 1 or 10ng/mL IL-10. TNFα secretion by macrophages in each condition was then assessed.


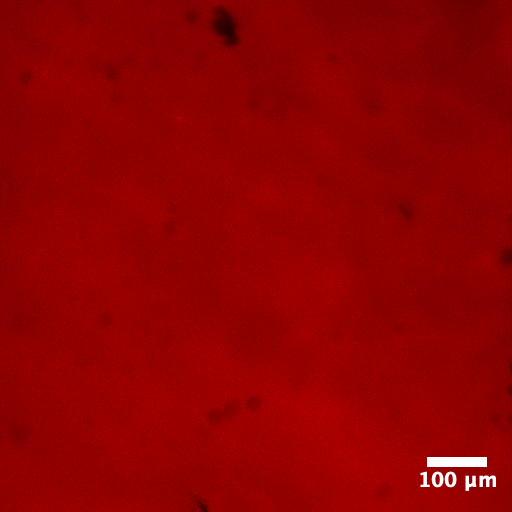

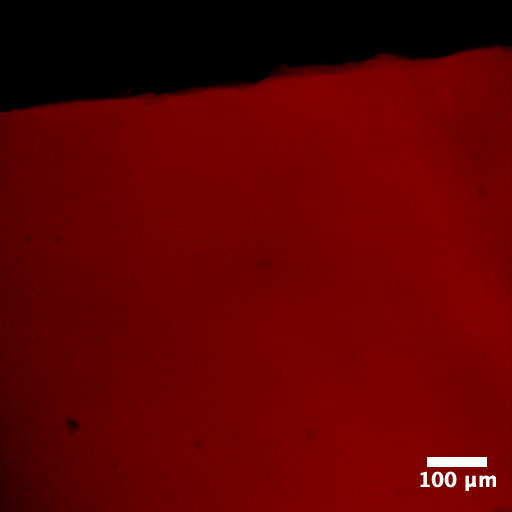

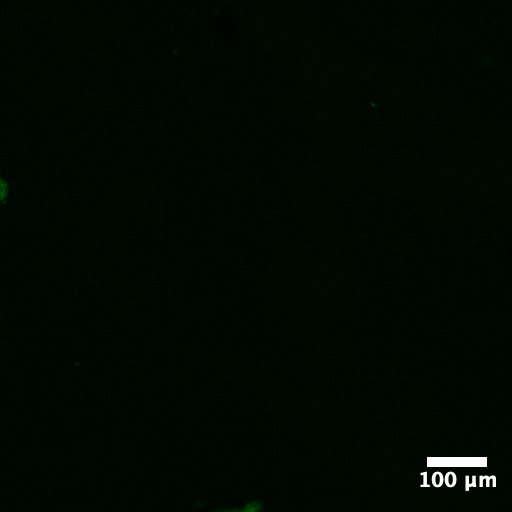

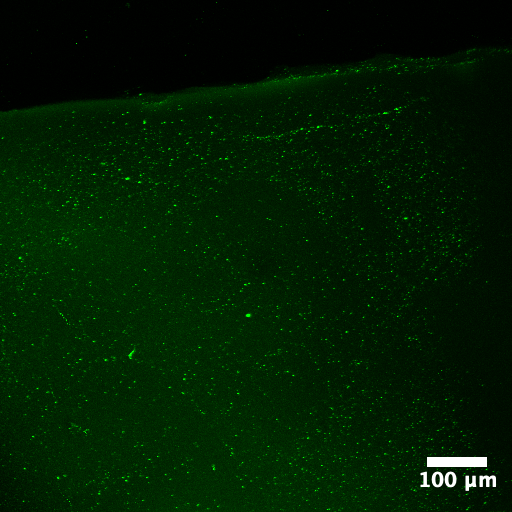


Unmodified IL-10

IL-10 N_3_

Hydrogel IL-10

**Figure S3: Azide-modified IL-10 can be tethered into hydrogel networks.** Bulk gels with excess DBCO groups were fabricated with AlexaFluor 647 (40μm) (red) and either unmodified IL-10 or IL-10-N_3_ at 10ng/mL. Gels were swollen in PBS for 24 hours, washed, blocked and stained for IL-10 (green).

**Figure S4: Azide modified IL-10 remains tethered to microgel scaffolds.** IL-10 or IL-10-N_3_ was included (10ng/mL) during microgel formation and microgels were assembled into acellular scaffolds and placed in 1mL of THP-1 media. Media was collected at 24, 48, and 120 hours after scaffold formation and assessed for total IL-10 release.


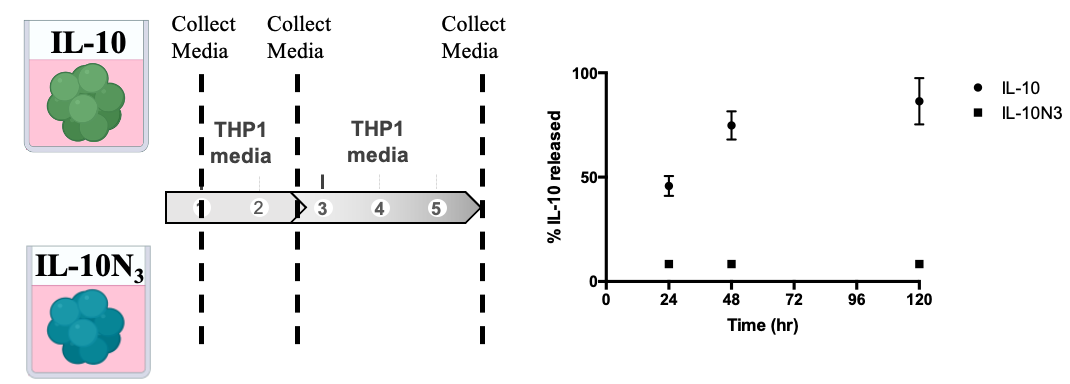

Supplement: Supplementary file 1 — Appendix S1: Supporting Information [file BTM2-6-e10217-s001.docx]
